# The association between the red blood cell distribution width-to-albumin ratio and female infertility: A cross-sectional study

**DOI:** 10.1097/MD.0000000000048002

**Published:** 2026-03-13

**Authors:** Qiuliang Yu, Yue Cao

**Affiliations:** a Department of Ultrasound, Jiaxing Hospital of Traditional Chinese Medicine, Jiaxing, Zhejiang, China; b Department of Cardiology, The Affiliated Hospital, Southwest Medical University, Luzhou, Sichuan, China.

**Keywords:** infertility, inflammation, NHANES, RAR, red blood cell distribution width to albumin ratio

## Abstract

Systemic inflammation and oxidative stress play key roles in female infertility. The red cell distribution width-to-albumin ratio (RAR) is a novel biomarker reflecting inflammation and nutritional status, but its association with female infertility remains unclear. This study explores the relationship between RAR and female infertility. Data from the 2013 to 2020 National Health and Nutrition Examination Survey were analyzed. Women aged 20 to 45 years with complete infertility and RAR data were included. Infertility was defined as unsuccessful conception after ≥12 months of attempts. RAR was calculated as red cell distribution width divided by albumin. Weighted logistic regression assessed the association between RAR and infertility, while restricted cubic spline regression examined nonlinear relationships. Subgroup and sensitivity analyses evaluated the robustness of the findings. A total of 4648 women were included. In the fully adjusted model, RAR was positively associated with infertility (odds ratio = 1.29, 95% confidence interval: 1.07–1.54, *P* = .01), with a significant trend test (*P* for trend < .05). Restricted cubic spline analysis indicated a primarily linear association (*P* for nonlinear > .05). Subgroup analyses showed consistent results, and propensity score matching confirmed the robustness of the findings. RAR is positively associated with female infertility, with consistent findings across subgroup and sensitivity analyses. As a biomarker of inflammation and nutritional status, RAR may have potential applications in reproductive health assessment.

## 1. Introduction

Infertility is a major global public health concern, affecting approximately 10% to 15% of women of reproductive age and imposing substantial physical, psychological, and socioeconomic burdens.^[1,2]^ Its etiology is multifactorial, involving reproductive endocrine dysfunction, metabolic disorders, chronic inflammation, and oxidative stress.^[3–7]^ Growing evidence suggests that systemic inflammation and nutritional status play critical roles in female reproductive health by influencing follicular development, ovulation, and embryo implantation, thereby increasing the risk of infertility.^[8–10]^ Identifying biomarkers that comprehensively reflect inflammation and nutritional status is essential for early screening and risk prediction of infertility.

Red cell distribution width (RDW) and albumin (ALB) are widely used hematological biomarkers for assessing inflammation, nutritional status, and systemic metabolism.^[11–13]^ RDW reflects red blood cell size variability and is influenced by inflammation, oxidative stress, and bone marrow function; its elevation has been linked to cardiovascular disease, diabetes, and chronic inflammatory conditions.^[14,15]^ ALB, a major plasma protein, not only indicates nutritional status but also possesses antioxidant, anti-inflammatory, and immunomodulatory properties.^[16]^ Recently, the red cell distribution width-to-albumin ratio (RAR), defined as RDW/ALB, has emerged as a composite biomarker that better reflects chronic inflammation and nutritional metabolism. It has shown predictive value in cardiovascular disease, diabetes, and other chronic conditions,^[17,18]^ and has also been linked to chest pain and urinary incontinence in recent population-based studies.^[19,20]^

Despite the potential impact of inflammation and nutrition on female fertility, epidemiological evidence on the association between RAR and infertility remains limited. This study utilizes data from the 2013 to 2020 National Health and Nutrition Examination Survey (NHANES) to examine the relationship between RAR and female infertility. Weighted multivariable logistic regression was used to assess the association's strength, restricted cubic spline (RCS) analysis evaluated potential nonlinear relationships, and propensity score matching (PSM) was performed for sensitivity analysis. Findings from this study may help elucidate the role of RAR in reproductive health and provide new biological insights for early infertility identification.

## 2. Materials and methods

### 2.1. Survey description

This study utilized NHANES (2013–2020), a nationally representative cross-sectional survey assessing health and nutrition in the U.S. NHANES employs a multistage, stratified probability sampling approach, collecting data via household interviews, physical exams at mobile examination centers, and laboratory tests. The NCHS Ethics Review Board approved all study protocols, and participants provided informed consent.

### 2.2. Study population

**Figure 1.**
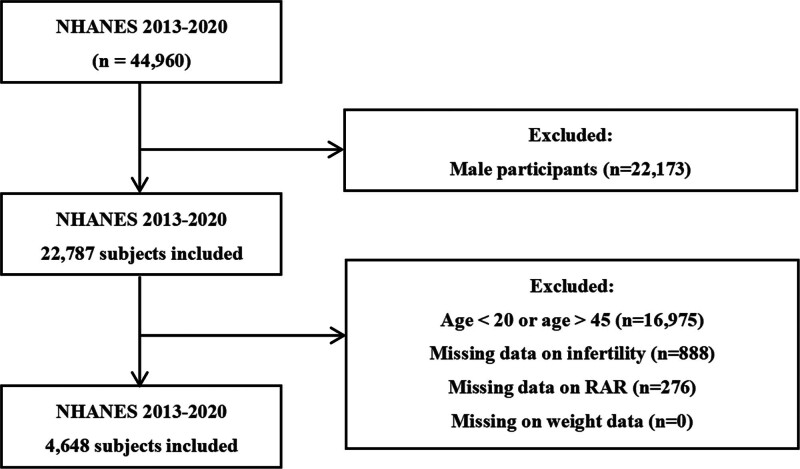
Flowchart of participant selection.

Figure 1 outlines the selection process. Of 44,960 NHANES participants, 22,173 men were excluded, leaving 22,787 women. Additional exclusions included those aged <20 or >45 years (n = 16,975), missing infertility data (n = 888), missing RAR data (n = 276), and missing weight data (n = 0). The final analysis included 4648 women.

### 2.3. Infertility assessment

Infertility was determined using NHANES reproductive health question RHQ074: "Have you ever tried to get pregnant for at least a year without success?" A "yes" response classified participants as infertile.

### 2.4. RAR assessment

RAR was calculated as RDW/ALB. RDW was obtained via a complete blood count, while ALB was measured using automated biochemical analysis. NHANES ensured standardized procedures and strict quality control.

### 2.5. Covariates

Covariates were selected based on the literature and potential confounders,^[21]^ including: demographic factors (age, race: White, Black, Mexican, other); socioeconomic status (poverty income ratio [PIR]; education: above high school, high school, less high school; marital status: married, never married, separated); lifestyle factors (smoking: yes/no; alcohol use: yes/no); and health conditions (body mass index [BMI]: <25, ≥25 kg/m^2^; pregnancy history: yes/no; hypertension: yes/no; diabetes: yes/no).

### 2.6. Statistical analysis

All analyses accounted for the NHANES complex sampling design and applied sample weights to ensure nationally representative estimates. Baseline characteristics were reported as means (standard error) for continuous variables and percentages (95% confidence interval [CI]) for categorical variables. Group differences were assessed using weighted *t* tests (for continuous variables) and Chi-square tests (for categorical variables). Logistic regression models assessed the relationship between RAR and female infertility, reporting odds ratios (ORs) and 95% CIs. Four models were constructed: Model 1 (unadjusted), Model 2 (adjusted for age, race, PIR, education), Model 3 (further adjusted for BMI, smoking, alcohol), and Model 4 (fully adjusted for all covariates). A trend test was conducted to examine the association between RAR and infertility. Subgroup analyses were performed to assess consistency across different population groups. Sensitivity analysis using PSM was conducted to validate the robustness of the results. All statistical analyses were conducted using R version 4.1.0 (R Foundation for Statistical Computing, Vienna, Austria), with a two-sided *P*-value <.05 considered statistically significant.

## 3. Results

### 3.1. Baseline characteristics

Table 1 presents the weighted characteristics of participants by RAR quartiles. Higher RAR was associated with older age (*P* < .001) and lower PIR (*P* < .001). The proportion of White participants decreased, while Mexican Americans, Blacks, and other races increased with higher RAR (*P* < .001). BMI ≥25 was more common in higher RAR groups (*P* < .001). Lower education levels (high school or below) were more frequent in higher RAR groups (*P* < .001). Marital status showed no significant difference (*P* = .33). Pregnancy history (*P* < .001), hypertension (*P* < .001), and diabetes (*P* < .001) were more prevalent in higher RAR groups. Infertility rates were significantly elevated in the highest RAR group (*P* < .001), while smoking (*P* = .78) and alcohol use (*P* = .09) showed no significant variation.

**Table 1:** Weighted baseline characteristics of participants.

Variables	Q1 N = 1168	Q2 N = 1150	Q3 N = 1167	Q4 N = 1163	*P* value
RAR	2.87 (0.01)	3.18 (0.00)	3.50 (0.00)	4.29 (0.02)	<.001
Age	31.29 (0.32)	32.03 (0.27)	33.20 (0.26)	33.58 (0.33)	<.001
PIR	3.02 (0.08)	2.70 (0.07)	2.56 (0.08)	2.33 (0.08)	<.001
Race					<.001
Black	77 (3.38)	199 (9.68)	328 (16.84)	463 (26.67)	
Mexican	189 (9.48)	188 (11.17)	201 (13.38)	187 (14.47)	
Other	385 (17.83)	339 (17.37)	298 (20.16)	279 (20.23)	
White	517 (69.30)	424 (61.79)	340 (49.62)	234 (38.63)	
BMI					<.001
<25	632 (54.46)	432 (37.00)	286 (24.89)	166 (15.30)	
≥25	536 (45.54)	718 (63.00)	881 (75.11)	997 (84.70)	
Education					<.001
Above high school	847 (77.52)	785 (73.02)	737 (65.20)	660 (55.99)	
High school	177 (14.90)	191 (16.89)	252 (22.90)	279 (27.20)	
Less high school	144 (7.58)	174 (10.10)	178 (11.90)	224 (16.80)	
Marital status					.33
Married	690 (60.90)	649 (57.08)	652 (57.51)	658 (60.87)	
Never married	365 (29.72)	372 (32.67)	380 (30.72)	366 (28.12)	
Separated	113 (9.38)	129 (10.25)	135 (11.77)	139 (11.00)	
Pregnancy					<.001
No	148 (13.76)	131 (13.30)	124 (10.50)	83 (6.83)	
Yes	1020 (86.24)	1019 (86.70)	1043 (89.50)	1080 (93.17)	
Hypertension					<.001
No	1057 (90.07)	988 (87.58)	999 (86.84)	914 (80.42)	
Yes	111 (9.93)	162 (12.42)	168 (13.16)	249 (19.58)	
Diabetes					<.001
No	1152 (99.21)	1100 (96.45)	1102 (94.74)	1082 (93.61)	
Yes	16 (0.79)	50 (3.55)	65 (5.26)	81 (6.39)	
Smoking					.78
No	846 (68.28)	814 (67.96)	804 (66.53)	805 (66.11)	
Yes	322 (31.72)	336 (32.04)	363 (33.47)	358 (33.89)	
Alcohol use					.09
No	174 (10.83)	157 (10.42)	180 (13.41)	194 (13.54)	
Yes	994 (89.17)	993 (89.58)	987 (86.59)	969 (86.46)	
Infertility					<.001
No	1050 (89.78)	1031 (89.80)	1022 (86.92)	992 (83.08)	
Yes	118 (10.22)	119 (10.20)	145 (13.08)	171 (16.92)	

BMI = body mass index, PIR = poverty income ratio, RAR = ratio of red blood cell distribution width to albumin level.

### 3.2. Association between RAR and female infertility

Table 2 shows that the association between RAR and infertility remained consistent across all models. In the fully adjusted model (Model 4), RAR was significantly associated with female infertility (OR = 1.29, 95% CI: 1.07–1.54, *P* = .01). Quartile-based analysis indicated that women in the highest RAR quartile (Q4) had a significantly higher risk of infertility compared to those in the lowest quartile (Q1) (OR = 1.51, 95% CI: 1.07–2.15, *P* = .02). The trend test was significant across all models (*P* = .004), supporting a positive association between RAR and infertility.

**Table 2:** Association between RAR and female infertility.

Exposures	Model 1	Model 2	Model 3	Model 4
RAR (continuous)	1.40 (1.19, 1.65) <.001	1.38 (1.17, 1.63) <.001	1.32 (1.10, 1.57) .003	1.29 (1.07, 1.54) .01
RAR (quartiles)	–	–	–	–
Q1	Ref	Ref	Ref	Ref
Q2	1.00 (0.72, 1.39) .99	0.98 (0.70, 1.37) .90	1.17 (0.86, 1.59) .30	0.93 (0.66, 1.30) .66
Q3	1.32 (0.97, 1.80) .08	1.28 (0.93, 1.76) .12	1.57 (1.13, 2.20) .01	1.17 (0.85, 1.60) .33
Q4	1.79 (1.29, 2.49) <.001	1.77 (1.27, 2.46) .001	1.17 (0.86, 1.59) .30	1.51 (1.07, 2.15) .02
*P* for trend	<.001	<.001	<.001	.004

Model 1: non-adjusted.

Model 2: adjusted for age, race, education, and PIR.

Model 3: further adjusted for BMI, smoking, and alcohol use based on Model 2.

Model 4: further adjusted for pregnancy, marital status, hypertension, and diabetes based on model.

BMI = body mass index, PIR = poverty income ratio, RAR = red blood cell distribution width-to-albumin ratio.

**Figure 2.**
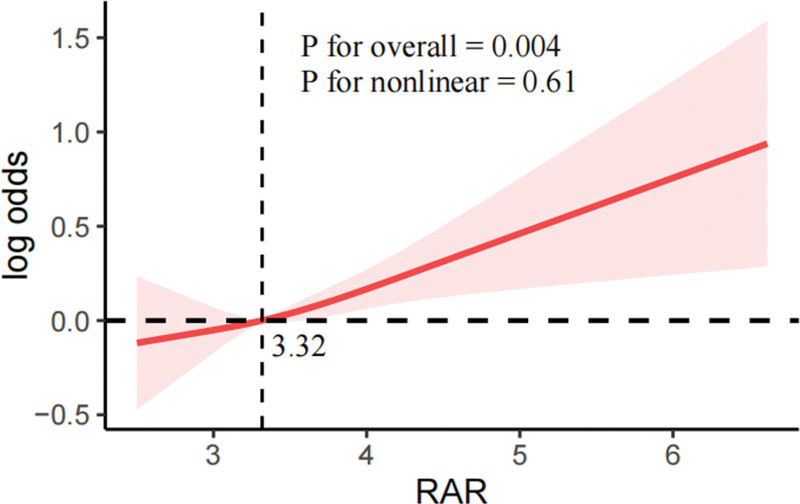
Nonlinear relationship between RAR and female infertility. RAR = red blood cell distribution width-to-albumin ratio.

Figure 2 illustrates the nonlinear association between RAR and infertility. The overall trend was significant (*P* for overall = .004), but the nonlinear test was not (*P* for nonlinear = .61), suggesting a predominantly linear relationship. A threshold analysis indicated that infertility risk increased significantly when RAR exceeded 3.32.

### 3.3. Subgroup analysis

**Figure 3.**
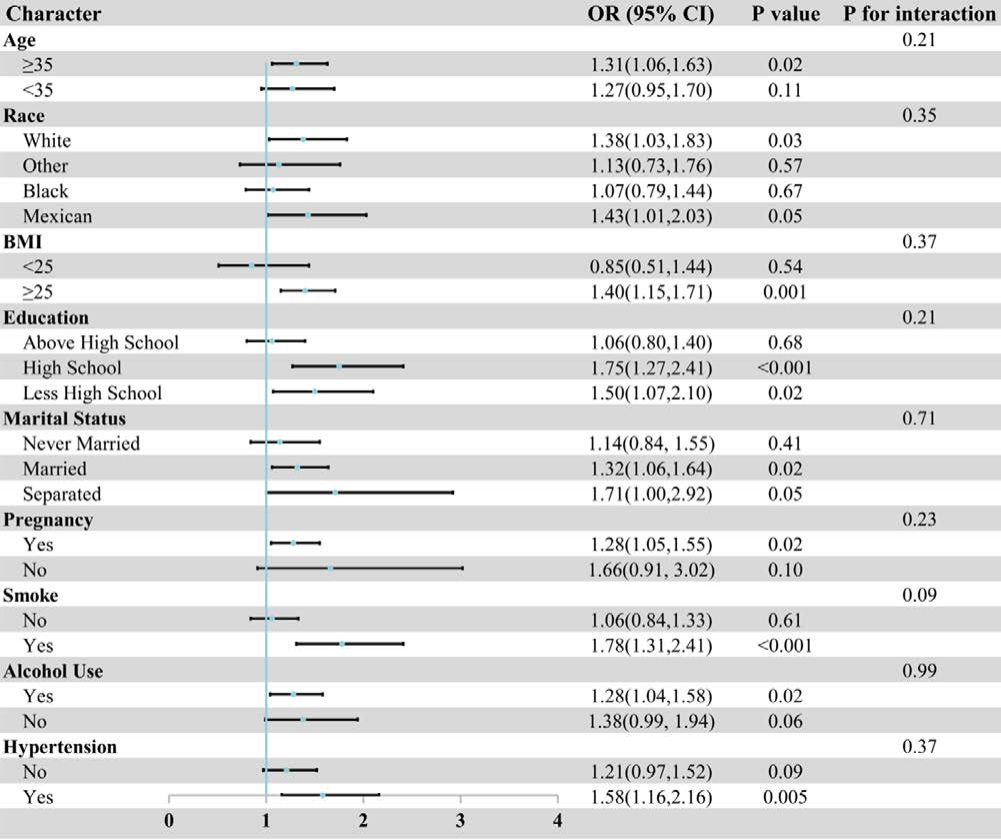
Association between RAR and infertility across subgroups. RAR = red blood cell distribution width-to-albumin ratio.

Figure 3 displays the association between RAR and infertility across subgroups. Most interaction tests (*P* for interaction) were not statistically significant, indicating consistent associations across different populations. In age-stratified analysis, the association was more pronounced in women ≥35 years (OR = 1.31, *P* = .02) than those <35 years (OR = 1.27, *P* = .11). The association was also stronger among women with lower education (OR = 1.50, *P* = .02) and those who had ever been pregnant (OR = 1.28, *P* = .02), although no significant interactions were observed. Overall, the positive association between RAR and infertility remained stable across subgroups, with no notable differences between groups.

### 3.4. Sensitivity analysis

Table 3 presents the characteristics after PSM. Following matching, there were no significant differences in covariates between the infertility group (n = 553) and the control group (n = 553) (*P* > .05), confirming effective balancing. However, the positive association between RAR and infertility remained. This further supports the robustness of the RAR-infertility association, independent of major confounders.

**Table 3:** Weighted baseline characteristics of matched participants.

Variables	Controls (n = 553)	Infertility (n = 553)	*P* value
Age	34.31 (0.32)	34.96 (0.41)	.22
PIR	2.91 (0.09)	2.76 (0.10)	.21
RAR	3.40 (0.03)	3.50 (0.04)	.02
Race			.99
Black	120 (10.93)	123 (12.45)	
Mexican	84 (10.01)	82 (10.13)	
Other	143 (16.29)	142 (16.32)	
White	206 (62.77)	206 (61.09)	
BMI			.83
<25	139 (29.80)	143 (25.87)	
≥25	414 (70.20)	410 (74.13)	
Education			.82
Above high school	358 (69.85)	363 (68.61)	
High school	103 (18.20)	106 (20.36)	
Less high school	92 (11.95)	84 (11.03)	
Marital status			.7
Married	383 (70.16)	386 (74.68)	
Never married	89 (15.04)	97 (14.34)	
Separated	81 (14.80)	70 (10.98)	
Pregnancy			.72
No	61 (11.86)	56 (14.07)	
Yes	492 (88.14)	497 (85.93)	
Hypertension			.48
No	458 (86.04)	448 (81.38)	
Yes	95 (13.96)	105 (18.62)	
Diabetes			.74
No	508 (95.04)	505 (91.69)	
Yes	45 (4.96)	48 (8.31)	
Smoking			.55
No	340 (58.60)	351 (60.51)	
Yes	213 (41.40)	202 (39.49)	
Alcohol use			.58
No	76 (11.24)	69 (9.50)	
Yes	477 (88.76)	484 (90.50)	

BMI = body mass index, PIR = poverty income ratio, RAR = red blood cell distribution width-to-albumin ratio.

## 4. Discussion

This study, using 2013 to 2020 NHANES data, is the first to investigate the association between RAR and female infertility. The results indicate that after adjusting for potential confounders, RAR remains significantly positively associated with infertility, with trend tests confirming the stability of this association. Additionally, RCS analysis suggests a primarily linear relationship, implying that higher RAR levels may progressively increase infertility risk. PSM analysis further supports the robustness of these findings.

As a composite biomarker, RAR reflects systemic inflammation, oxidative stress, and nutritional status. In this study, higher RAR was linked to increased infertility risk, suggesting that chronic inflammation, oxidative stress, and nutritional imbalance may contribute to infertility. Elevated RDW, a key component of RAR, is associated with inflammation and oxidative stress, which can impair ovarian function.^[22]^ RDW reflects red blood cell volume variability and is influenced by inflammation, oxidative stress, and bone marrow function.^[23–25]^ Increased RDW suggests impaired erythropoiesis, delayed maturation, or accelerated destruction and is strongly linked to chronic inflammatory diseases such as cardiovascular disease, diabetes, and metabolic syndrome.^[26,27]^ In the reproductive system, inflammatory mediators (e.g., interleukin-6, tumor necrosis factor alpha, and interleukin-1β) may disrupt follicular development, corpus luteum function, and endometrial receptivity, thereby increasing infertility risk.^[28–31]^ Additionally, oxidative stress can lead to mitochondrial damage, DNA fragmentation, and apoptosis in ovarian cells, ultimately reducing oocyte quality and reproductive outcomes.^[32–34]^ Low ALB levels, another component of RAR, may indicate chronic inflammation, malnutrition, liver dysfunction, and increased oxidative stress.^[35–37]^ A higher RAR, characterized by elevated RDW and reduced ALB, likely represents more severe systemic inflammation, thereby exacerbating infertility risk.

In addition to reproductive outcomes, accumulating clinical and epidemiological evidence suggests that RAR is associated with a broad spectrum of disease outcomes. Elevated RAR has been linked to a higher prevalence of chest pain and urinary incontinence, extending its clinical relevance beyond reproductive health.^[19,20]^ Beyond symptom-based outcomes, RAR has been shown to independently predict all-cause mortality in critically ill patients with nonalcoholic fatty liver disease and to be associated with increased risk of cause-specific mortality (including cardiovascular, respiratory, and diabetic causes) in general population cohorts.^[18,38]^ Moreover, higher RAR has been linked to poorer prognosis in chronic obstructive pulmonary disease patients and other chronic disease settings, with a generally consistent direction of association indicating greater disease burden at higher RAR levels.^[39–41]^ These findings support the broader clinical relevance of RAR as a nonspecific marker of chronic inflammatory burden and provide additional context for its observed association with female infertility.

While no prior studies have directly examined RAR and infertility, existing literature has reported the roles of RDW and ALB in reproductive health. Elevated RDW has been associated with adverse reproductive outcomes, including polycystic ovary syndrome, endometriosis, and hypertensive disorders of pregnancy. For instance, studies have shown that RDW levels are significantly higher in women with polycystic ovary syndrome than in healthy controls and correlate positively with insulin resistance, inflammatory markers, and androgen levels, suggesting that RDW may influence ovarian function through inflammatory and metabolic pathways.^[42,43]^ Moreover, RDW has been linked to the severity of endometriosis, potentially affecting fertility by altering endometrial cell proliferation and immune regulation.^[44,45]^ In pregnancy-related conditions, elevated RDW is associated with placental dysfunction and adverse pregnancy outcomes, such as preterm birth and fetal growth restriction, further highlighting its potential impact on female reproductive health.^[46–48]^ Additionally, low ALB levels have been linked to an imbalance in follicular fluid prostanoids, which may play a role in maintaining oocyte quality.^[49]^ This study extends existing evidence by demonstrating a significant positive association between RAR and infertility, proposing RAR as a novel predictive biomarker.

RCS analysis further confirms the linear association between RAR and infertility, with an inflection point at RAR >3.32, beyond which infertility risk rises significantly. This threshold effect suggests that once RAR surpasses a certain level, its impact on infertility may intensify, potentially due to cumulative inflammatory and oxidative stress effects. Subgroup analysis showed that this association remained consistent across different population groups, with no significant interactions detected, further reinforcing the robustness of the findings. Additionally, PSM results were consistent with the primary analysis, confirming that the association between RAR and infertility is independent of major confounders.

This study has several strengths. First, the use of NHANES data, with its large sample size and nationally representative population, enhances the generalizability of the findings. Second, multiple analytical approaches, including RCS, subgroup analysis, and PSM, were employed to ensure the robustness of the results and minimize confounding effects. Additionally, RAR is a readily available, cost-effective, and reproducible biomarker, which may have practical implications for clinical infertility risk assessment. However, certain limitations should be acknowledged. First, the cross-sectional design of NHANES prevents the establishment of causality; thus, longitudinal or mechanistic studies are needed to confirm the findings. Second, infertility assessment was based on self-reported data, which may introduce recall bias. Nonetheless, NHANES questionnaires have been widely validated and used in epidemiological studies. Third, although multiple confounders were adjusted for, residual confounding due to unmeasured factors such as inflammatory markers, dietary patterns, and lifestyle habits cannot be ruled out. Future research should further explore these factors.

## 5. Conclusions

This study provides the first evidence of a positive association between RAR and female infertility, suggesting that higher RAR may indicate an increased risk of infertility. As a biomarker reflecting both inflammation and nutritional status, RAR may have potential applications in reproductive health assessment. Future prospective cohort and longitudinal studies are warranted to clarify the temporal and potential causal relationship between RAR and infertility, to elucidate the underlying biological mechanisms, and to evaluate whether interventions targeting inflammation and nutritional status could reduce infertility risk.

## Acknowledgments

The authors acknowledge the use of ChatGPT (OpenAI, San Francisco) exclusively for language editing. The authors are fully responsible for the content of this work.

## Author contributions

**Conceptualization:** Qiuliang Yu.

**Data curation:** Qiuliang Yu.

**Formal analysis:** Qiuliang Yu.

**Methodology:** Yue Cao.

**Software:** Yue Cao.

**Validation:** Yue Cao.

**Writing – original draft:** Qiuliang Yu.

**Writing – review & editing:** Yue Cao.
